# On the Evolution of *Yeti*, a *Drosophila melanogaster* Heterochromatin Gene

**DOI:** 10.1371/journal.pone.0113010

**Published:** 2014-11-18

**Authors:** Roberta Moschetti, Emanuele Celauro, Fulvio Cruciani, Ruggiero Caizzi, Patrizio Dimitri

**Affiliations:** 1 Dipartimento di Biologia, Università degli Studi di Bari, Bari, Italy; 2 Dipartimento di Biologia e Biotecnologie “Charles Darwin” and Istituto Pasteur Fondazione Cenci-Bolognetti, Sapienza Università di Roma, Roma, Italy; Virginia Tech, United States of America

## Abstract

Constitutive heterochromatin is a ubiquitous and still unveiled component of eukaryotic genomes, within which it comprises large portions. Although constitutive heterochromatin is generally considered to be transcriptionally silent, it contains a significant variety of sequences that are expressed, among which about 300 single-copy coding genes have been identified by genetic and genomic analyses in the last decades. Here, we report the results of the evolutionary analysis of *Yeti*, an essential gene of *Drosophila melanogaster* located in the deep pericentromeric region of chromosome 2R. By FISH, we showed that *Yeti* maintains a heterochromatin location in both *D. simulans* and *D. sechellia* species, closely related to *D. melanogaster*, while in the more distant species e.g., *D. pseudoobscura* and *D. virilis*, it is found within euchromatin, in the syntenic chromosome Muller C, that corresponds to the *2R* arm of *D. melanogaster* chromosome 2. Thus, over evolutionary time, *Yeti* has been resident on the same chromosomal element, but it progressively moved closer to the pericentric regions. Moreover, *in silico* reconstruction of the *Yeti* gene structure in 19 *Drosophila* species and in 5 non-drosophilid dipterans shows a rather stable organization during evolution. Accordingly, by PCR analysis and sequencing, we found that the single intron of *Yeti* does not undergo major intraspecies or interspecies size changes, unlike the introns of other essential *Drosophila* heterochromatin genes, such as *light* and *Dbp80.* This implicates diverse evolutionary forces in shaping the structural organization of genes found within heterochromatin. Finally, the results of d_S_ - d_N_ tests show that *Yeti* is under negative selection both in heterochromatin and euchromatin, and indicate that the change in genomic location did not affected significantly the molecular evolution of the gene. Together, the results of this work contribute to our understanding of the evolutionary dynamics of constitutive heterochromatin in the genomes of higher eukaryotes.

## Introduction

Constitutive heterochromatin is commonly found in large blocks near centromeres and telomeres; it consists mostly of repetitive DNA sequences and maintains its characteristic organization on both homologous chromosomes. It is a ubiquitous component of eukaryotic genomes and, in many species, comprises large chromosomal portions, or even entire chromosomes. For example, about 30% of the *Drosophila* and human genomes, and up to 70–90% of certain nematode and plant genomes, are made up of constitutive heterochromatin [1], [2], [3], yet the reasons for its widespread occurrence are still unclear.

Heterochromatin was originally defined at cytological level as the chromosome portion that stains deeply at prophase and maintains a compact organization throughout all stages of the mitotic cell cycle [4]. Historically, distinctive antagonistic properties compared to the rest of the genome were identified: 1) strongly reduced level of meiotic recombination; 2) low gene density; 3) mosaic inactivation of the expression of euchromatic genes when moved nearby (position effect variegation, PEV); 4) late replication during S phase; 5) transcriptional inactivity; 6) enrichment in the so-called “junk” repetitive DNA, such as satellite sequences and truncated transposable element remnants.

Together, these properties led to the view of constitutive heterochromatin as a “desert” of genetic functions [5]. In the last three decades, however, studies primarily conducted in *Drosophila melanogaster* have shown that constitutive heterochromatin does in fact play roles in important cellular functions, such as chromosome organization and inheritance [6], [7], [8], [9], [10], [11]. Although generally regarded as transcriptionally silent, constitutive heterochromatin has been found to contain actively transcribed genes [3]. For example, in *Drosophila melanogaster*, more than 40 genes essential for viability or fertility have been mapped to pericentric heterochromatin [12], [13], [14], [15], [16], [17].

In the last decade, the release of *D. melanogaster* heterochromatin sequence by the Berkeley Drosophila Genome Project (http://www.fruitfly.org/) and Drosophila Heterochromatin Genome Project (http://www.dhgp.org/index_release_notes.html) has greatly facilitated studies of mapping, molecular organization and function of genes located in pericentromeric heterochromatin [18].

More recently, an improved whole genome shotgun assembly [19] has been produced, which includes 20.7 Mb of draft-quality heterochromatin sequence. In the last years, 15 Mb of this sequence have been further improved or completed [20] and a BAC-based physical map of 13 Mb of pericentric heterochromatin, together with the cytogenetic map that locates some 11 Mb to specific heterochromatin regions, have been constructed [20]. About 250 protein-coding genes were defined in the release 5.1 annotation of the currently sequenced heterochromatin DNA [21]. According to these results, the number of active genes in constitutive heterochromatin of *D. melanogaster* appears to be higher than defined by genetic analysis. Notably, these genes encode proteins involved in important cellular and developmental processes [3].

Further mapping of *D. melanogaster* heterochromatin was performed by comparative genomic hybridization [22]. The transcription profiles of mapped sequences by microarray analysis also revealed region-specific temporal patterns of transcription within heterochromatin during oogenesis and in early embryonic development.

Evolutionary studies have shown that *D. melanogaster* heterochromatin genes, such as *light* and others, originated from progenitors that were originally located within euchromatin in the drosophilid lineage [23], [24]. Here we have focussed our study on the evolutionary origin of *Yeti*, an essential heterochromatin gene of *D. melanogaster*, which encodes a protein belonging to the evolutionarily conserved BCNT family of chromatin remodellers [25], [26]. We report that *Yeti* locates in euchromatin in distant species, e.g. *D. pseudoobscura* and *D. virilis*, similarly to what has been found for *light* and other genes [23], [24]. Moreover, we found that the *Yeti* gene structure remains rather stable during the evolution of *Drosophila* species. In particular, the second exon that encodes the last 30 aminoacids of the conserved BCNT domain is invariably 91 bp-long. Finally, we found that the single intron of *Yeti* does not undergo major size changes in *D. melanogaster* and closely related species, unlike the introns of other essential *Drosophila* heterochromatin genes [27].

## Results

### Evolutionary repositioning of the *Yeti* gene from euchromatin to pericentric heterochromatin

**Figure 1:**
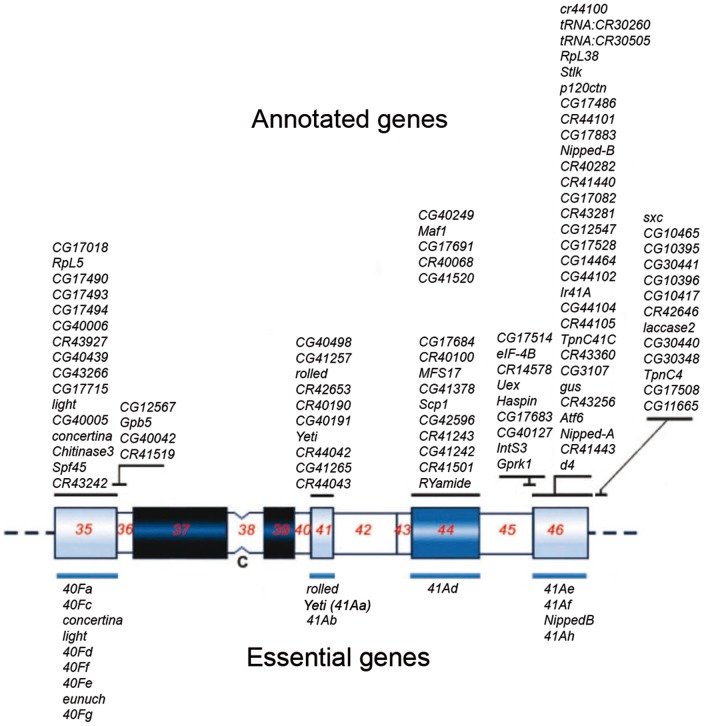
Cytogenetic mapping of heterochromatin genes of chromosome 2. The map was modified from that shown in previous papers [3], [45]. The diagram shows the essential genes defined by mutational analyses (below) and annotated genes defined by the heterochromatin genome project (above). Shades of blue correspond to the intensity of DAPI staining, with the darkest blue blocks representing regions with strong fluorescence intensity and open blocks representing non fluorescent regions. The different cytological regions are numbered.

The single-copy *Yeti* gene of *D. melanogaster* maps to the region h41 of chromosome 2R mitotic heterochromatin (Figure 1; Table 1), which corresponds to division 41A of salivary gland polytene chromosomes [25], [26], [28], [29].

**Table 1:** List of the *Yeti* orthologs and their encoded proteins.

Species	Gene ID	Database Location	GenBank A.C. (position)	Uniprot reference	Amino acids
***D.melanogaster***	FBgn0128734	2Rh:1343403..1345119	NW_001848856.1	B4J7U2	241
***D.simulans***	FBgn0191193	3R:38,269..39,056 [+]	NT_167061.1	B4QUX4	241
***D.sechellia***	FBgn0166002	2h;scaffold_170:38,673..3,460 [−].	NW_001999858.1	B4IME9	241
***D.yakuba***	FBgn0236265	2h;v2_chr2h_random_005:668,463..673,172.	NW_002052891.1	B4IT75	215
***D.erecta***	FBgn0103193	scaffold 4929	NW_001956548.1	B3N457	236
***D.eugracilis***	not available	not available	KB464511.1 (19716..18949)	not available	235
***D.biarmipes***	not available	not available	KB462255.1 (18839..19580)	not available	229**
***D.takahashi***	not available	not available	KB460683.1 (11044..10283)	not available	235**
***D.elegans***	not available	not available	KB458177.1 (6204..6991)	not available	244**
***D.ananassae***	FBgn0088065	2R euchromatic region scaffold 13266	NW_001939294.1	B3MBR1^a^	272^a^
***D.bipectinata***	not available	not available	KB464371.1 (48012..48773)	not available	232
***D.pseudoobscura***	FBgn0245984	2R euchromatic region	NC_009006.2	B5E0W2	275
***D.persimilis***	FBgn0148669	not available	NW_001985955.1	B4GBJ4	275
***D.miranda***	not available	not available	CM001519.2 (366522..367496)	not available	275
***D.willistoni***	FBgn0212772	2R euchromatic arm	NW_002032340.1	B4MJ08	273
***D.mojavensis***	FBgn0143134	2R euchromatic arm	NW_001979114.1	B4KQ8	295
***D.virilis***	FBgn0209341	2R euchromatic arm	NW_002014420.1	B4LKP7	300
***D.albomicans***	not available	not available	JH859027.1 (5894805..5895706)	not available	279
***D.grimshawi***	FBgn0128734	2R euchromatic arm	NW_001961673.1	B4J7U2	285
***C.quinquefasciatus***	CPIJ018830	not available	NW_001888048.1	B0XH12	284
***A.aegypti***	AAEL007422	supercontig 1.255, euchromatic region 2p25 [46]	NW_001810963.1	Q0IF03	279
***A.darlingi***	not available	not available	ADMH02000690.1 (45710.. 44829)	not available	
***A.gambiae***	AGAP005152	2L:euchromatic region 21E	NT_078265.2	Q7PPY4	293
***M.destructor***	not available	not available	GL501532.1 (369211.. 368309)	not available	300

**ORF defective. Amino acids deducted from bestfit alignments.

a) Bestfit protein is 236 aminoacids long.

To characterize the chromosomal location of *Yeti* among *Drosophila* genus species, we performed fluorescent *in situ* hybridization (FISH) experiments on polytene chromosomes of *D. simulans, D. sechellia*, two sibling species of *D. melanogaster*, and on two distantly related species: *D. pseudoobscura*, belonging to the Sophophora subgenus and *D. virilis*, belonging to the *Drosophila* subgenus. These species cover nearly a 40 million years divergence time and thus represent a wide spectrum of the evolutionary history of *Yeti*.

To map *Yeti* in *D. simulans* and *D. sechellia*, we used the *D. melanogaster Yeti* cDNA probe (RE36623), while PCR species-specific probes were used in *D. pseudoobscura* and *D. virilis.* PCR probes were amplified over a less conserved region located outside the C-terminal BCNT coding domain of YETI protein (see Materials and Methods).

**Figure 2:**
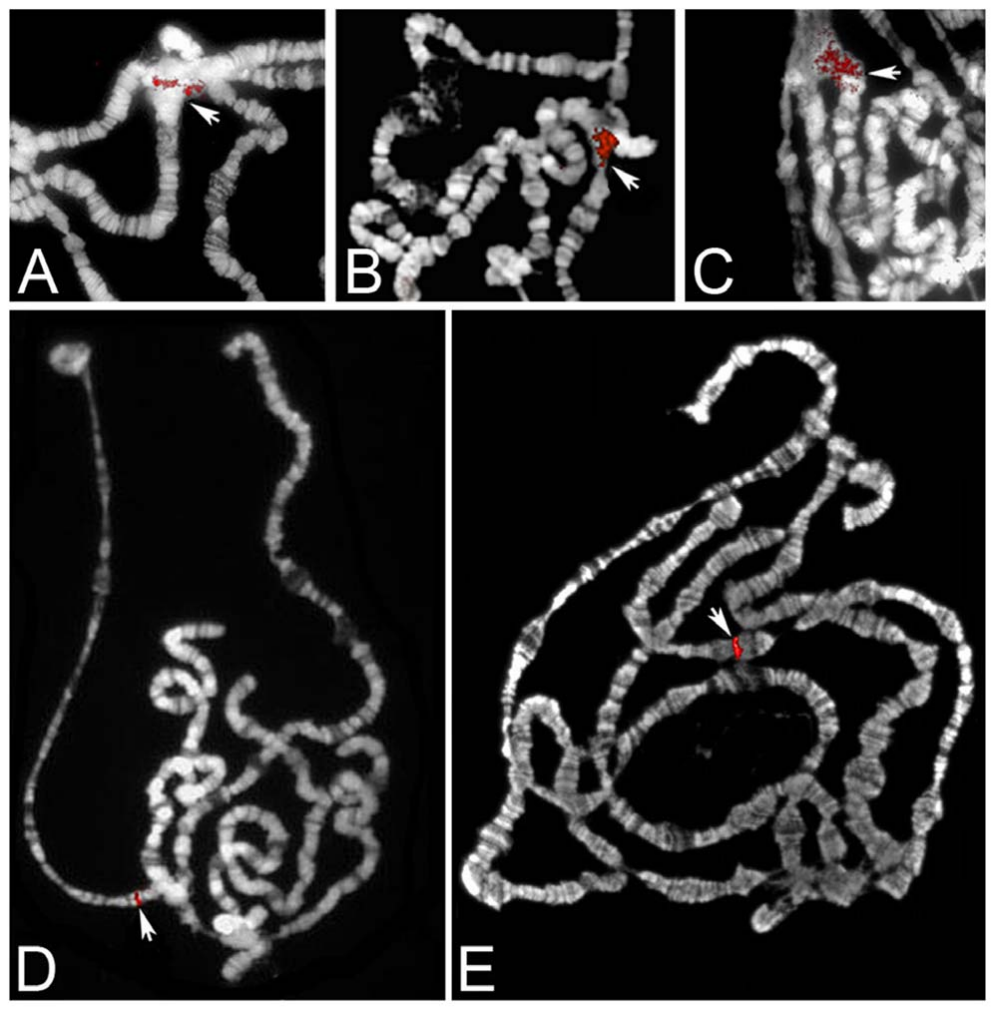
Examples of FISH mapping of *Yeti* probes to polytene chromosomes of *Drosophila* species. Salivary gland polytene chromosomes were stained with DAPI and pseudocolorated in blue; fluorescent signals were pseudocolorated in red. In *D. melanogaster* (A) and in the closely related *D. simulans* (B) and *D. sechellia* (C) species, the *Yeti* cDNA probe maps to *2Rh* at the base of polytene division 41. The large and diffuse morphology of the *Yeti* signal found in these species, reflects the disorganized and poorly banded structure of the heterochromatin in the chromocenter. The arrows point the base of *2Rh*. In *D. pseudobscura*, the hybridization signal of *Yeti* PCR probe maps to region 63C in the proximal euchromatin (D). In *D. virilis* the *Yeti* hybridization signal maps to region 53E, in the distal euchromatin of chromosome 5 (E).

The results of this analysis are shown in Figure 2. In *D. melanogaster, D. simulans* and *D. sechellia* the *Yeti* cDNA probe produces a signal mapping to the base of division 41A, in the right arm of chromosome 2 (Figure 2A,B,C). Notably, the signals show a large diffuse structure very different from the sharp hybridization signals usually seen with euchromatic probes; such a morphology is a distinctive mark for sequences derived from partially polytenized heterochromatin regions [30], [31]. Together, our FISH results indicate that *Yeti* maintains a heterochromatic location in *D. simulans* and *D. sechellia*. The FlyBase localization of *Yeti* in *D. sechellia* is in 2 h (scaffold_170:38,673..3,460; Table 1), in accord with our mapping results, while that in *D. simulans* in 3R (38,269..39,056; Table 1) is apparently conflicting and may reflect an assembly error in the *D. simulans* genome sequences (see discussion), as reported by Schaffer et al. [32]. In both *D. pseudoobscura and D. virilis* a single FISH signal was observed in the euchromatic arms of polytene chromosomes, in agreement with FlyBase (Figure 2D, E; Table 1). In *D. pseudoobscura*, the *Yeti PCR* probe produced a sharp signal that maps to region 63C in the proximal euchromatin of chromosome 3, while in *D. Virilis* the *Yeti* signal is found at region 53E, in the distal euchromatin of chromosome 5. Thus, independently of their genome localization (heterochromatin or euchromatin), in the analysed species *Yeti* lies in the syntenic chromosome Muller C, that corresponds to the *2R* arm of *D. melanogaster* chromosome 2.

### *In silico* reconstruction of *Yeti* gene organization in different sequenced genomes

To study the evolutionary conservation of the *Yeti* gene organization, we have characterized the structure of the *Yeti* orthologs in 19 *Drosophila* species (*D. melanogaster, D. simulans, D. sechellia, D. yakuba, D. erecta, D. eugracilis, D. biarmipes, D takahashii, D. elegans, D. ananassae, D. bipectinata, D. pseudobscura, D. persimilis, D. miranda, D. willistoni, D. mojavensisi, D. virilis, D. albomicans,* and *D. grimshawi*) and in five non-drosophilid dipterans (*C. quinquefasciatus, A. Aegypti, A. darlingi, A. gambiae* and *M. destructor* pest crop).

**Figure 3:**
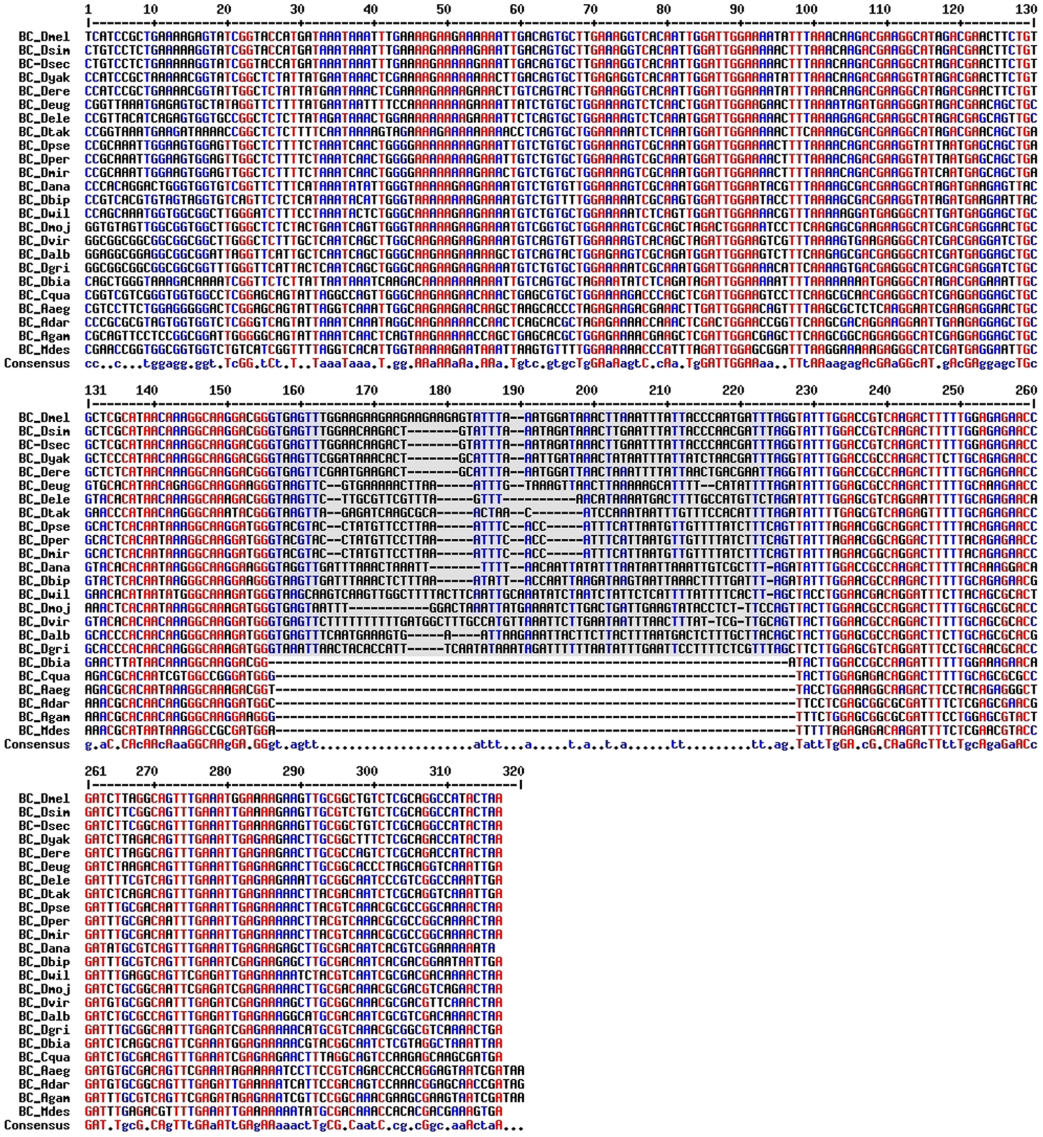
Alignment of the *Yeti* ortholog sequences encoding the BCNT-C domain. The grey area corresponds to the intron present in the *Drosophila* species.

All the *Yeti* DNA sequences were retrieved from FlyBase. In case of annotated genes, the YETI protein sequences were extracted from the Ortho DB database [33], where they are reported as orthologous sequences belonging to the BCNT family complex [26], [34]. For the recently sequenced genomes, the *Yeti* DNA sequences were recovered by the TblastN procedure, using the *D. melanogaster* YETI protein sequence. The alignments of the retrieved *Yeti* orthologs are shown in Figure 3 and their coordinates are reported in Table 1.

**Figure 4:**
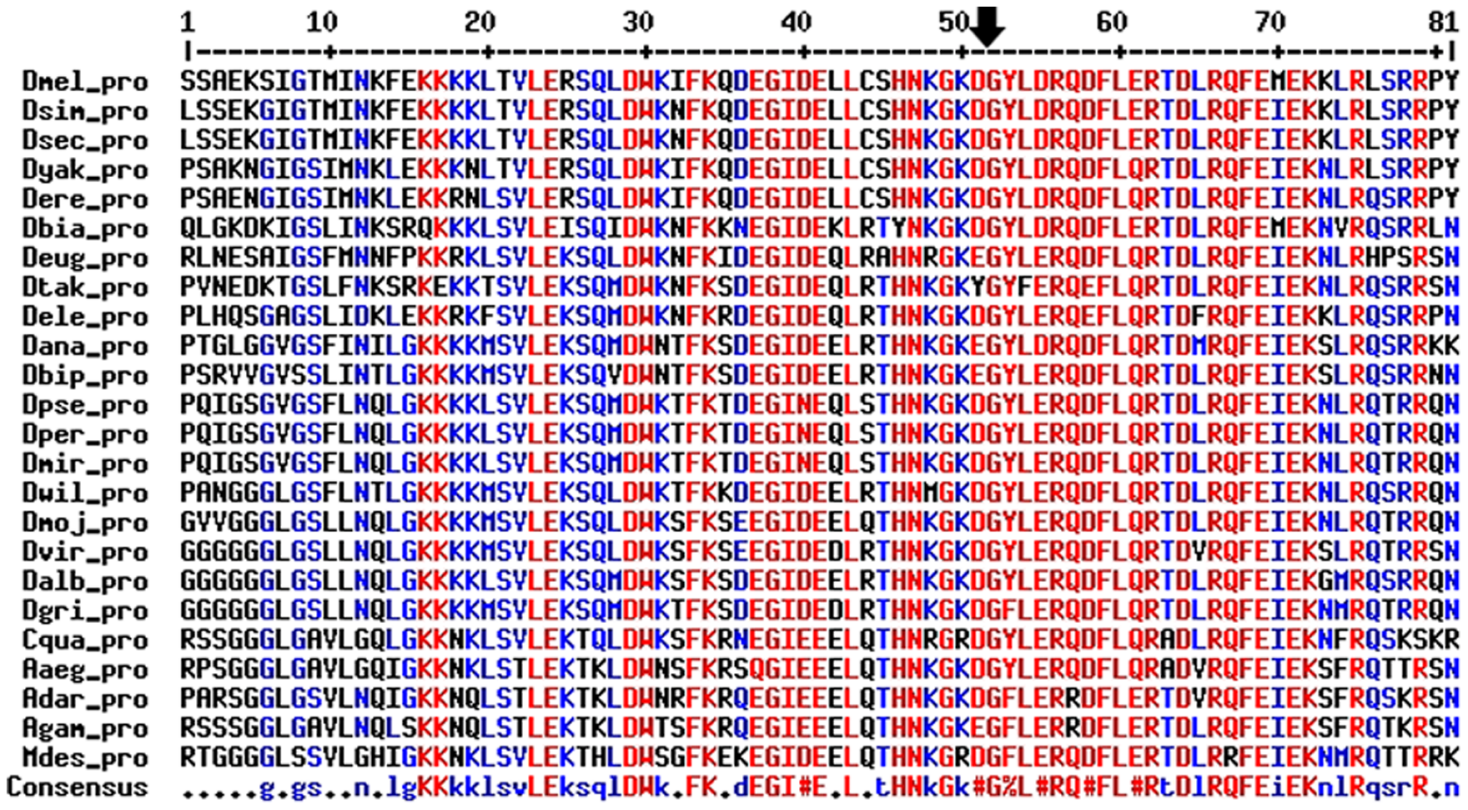
Alignment of the BCNT domain of YETI proteins among species. The arrow points the intron position in the corresponding coding region of *Drosophila* species.

We were able to recover a deducted complete protein sequence except for the recently added genomes of *D. biarmipes*, *D. takahashi* and *D. elegans*, where frame-shift mutations were found in the 5′ end of the gene, probably due to errors in sequencing that needs to be improved. However, in each case the 3′end of the gene, containing the BCNT domain-coding region, was detected (Figure 3). The protein sequence alignments show a strong conservation of the BCNT domain in the *Drosophila* genus and in non-drosophilid dipterans (Figure 4).

**Figure 5:**
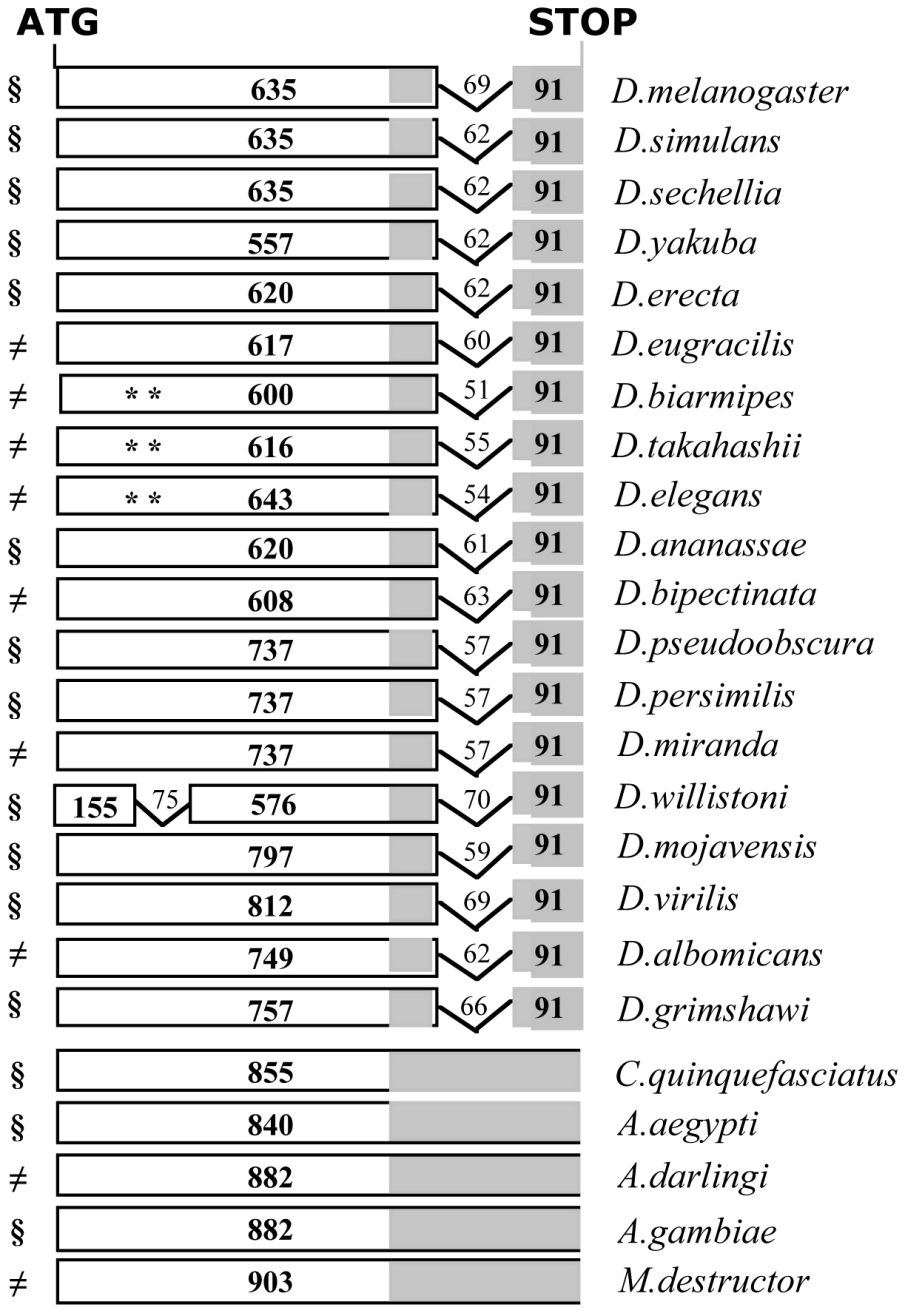
Comparison of the *Yeti* gene structure among sequenced genomes. Only the coding regions are showed. Exons are in boxes and numbers refer to nucleotides. Symbols: §, annotated genes; ≠, this study; **, defective ORF. The grey area at the 3′ end represents the conserved BCNT-C domain in the protein.

We next compared the reconstructed molecular organization of the *Yeti* gene in the above-mentioned species, to study whether it underwent substantial structural changes during evolution. The results of this analysis are shown in Figure 5. It appears that the structure of the *Yeti* orthologs, with two exons and one intron remained highly conserved during the divergence of the lineages in the *Drosophila* genus, the only exception being *D. willistoni*, where an additional intron of 75 bp is present. The gene size is identical among *D. melanogaster, D. simulans* and *D. sechellia*, the only detectable difference is represented by the intron that in *D. melanogaster* is 7 bp longer. In general, in the *Drosophila* genus both the first exon and the single intron undergo changes in size: the exon varies from 557 bp (*D. yakuba*) to 812 bp (*D. virilis*), while the intron spans from 51 bp (*D. biarmipes*) to 70 bp (*D. willistoni*). Notably, the size of the second exon, that encodes the last portion of the BCNT domain, shows a striking conservation in the *Drosophila* genus, being invariably 91 bp long. Finally, in the five genome species of non-drosophilid dipterans (*C. quinquefasciatus*, *A. Aegypti*, *A. darlingi*, *A. gambiae* and *M. destructor*) the organization of *Yeti* differs in that the intron disappears and a single exon is present.

### Characterization of the *Yeti* intron in *Drosophila* species

Intraspecific and interspecific size polymorphisms of heterochromatin gene introns have been found in *Drosophila*, which are likely to be associated with *de novo* insertions of TE-related sequences [27]. We then asked whether the intron of *Yeti* is prone to TE insertions or to other gross changes in length. To answer this question, we PCR amplified a region of about 180 bp comprising the *Yeti* intron from genomic DNAs extracted from *D. melanogaster* and *D. simulans* strains. Most of the analyzed strains derived from geographically distant natural populations (see materials and methods for the complete list). We also included in the analysis a single strain of *D. sechellia* and *D. teissieri*. The rationale of these experiments is that if an insertion have targeted the intron, it would have in turn increased the expected size of the amplified region.

**Figure 6:**
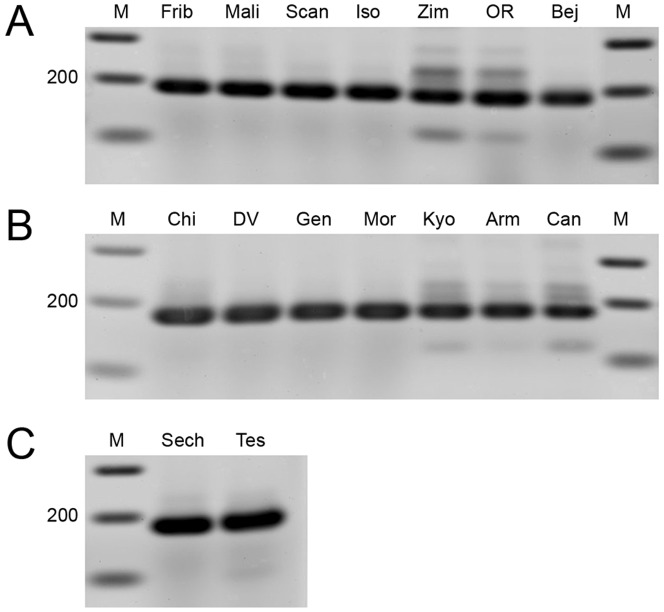
PCR amplification of the genomic region containing the *Yeti* intron in *Drosophila* species. A single PCR product of about 180 bp was found, in *D. melanogaster* (A) and *D. simulans*, (B) *D. sechellia* (C) and *D. tessieri* (C) related species. M =  Marker; Frib  =  Friburgo; Mal  =  Mali; Scan  = Scansano; Iso  =  *y^1^; cn^1^ bw^1^sp^1^* isogenic strain; OR  =  Oregon-R; Bej  =  Bejin; Chi  =  Chicharo; DV  =  Death Valley; Gen  =  Genoa; Mor  =  Moruya; Kyo  =  Kyogle; Arm  =  Armidale; Can  =  Canaries; Sech  =  *D. Sechellia*; tes  =  *D. tessieri*. Molecular weight is in bps.

**Figure 7:**
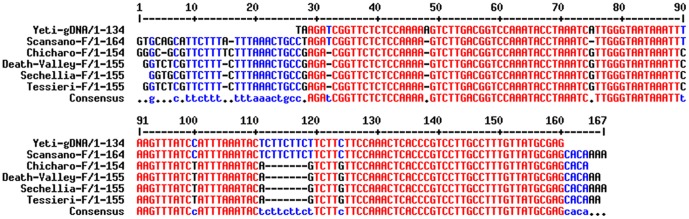
Sequencing of the purified PCR products from *Drosophila* species. Sequence alignments from Iso and Scansano (*D. melanogaster*), Chicharo and Death Valley (*D. Simulans*), *D. sechellia* and *D. tessieri*. Sequence analysis confirmed that they correspond to the *Yeti* intron containing region. The *Yeti* intron is shown in normal text, the flanking exons are in bold. The *D. simulans* and *D. sechellia* intron lacks a 7 bp stretch (see the gap), in agreement with the genome sequence data (see results and Figure 5). The *D. Tessieri* intron sequence is identical to that of *D. simulans* and *D. sechellia*.

We analysed 25 wild type strains and 4 laboratory stocks of *D. melanogaster*, as well as 9 wild type strains of *D. simulans* (see materials and methods). As shown in Figure 6, PCR amplification of *Yeti* produces a prominent band of the expected size in all the different strains of *D. melanogaster* (Figure 6A) and of *D. simulans* (Figure 6B) and in both *D. sechellia* and *D. tessieri* strains (Figure 6C). Sequencing of the purified PCR products from Iso and Scansano (*D. melanogaster*), Chicharo and Death Valley (*D. simulans*), *D. sechellia* and *D. tessieri* confirmed that they correspond to the *Yeti* intron-containing region (Figure 7). In conclusion, the results of this analysis suggest that the small intron of *Yeti* does not frequently undergo significant increase in size, unlike other essential heterochromatin genes of *Drosophila* [27]. This conclusion is in agreement with the observation that the *Yeti* gene structure tends to be stable during the evolution of the *Drosophila* genus, with the single intron that retains its short size (Figure 5).

### *Yeti* is under negative selection

We next asked whether *Yeti* has evolved under negative (purifying) or positive selection, and whether the change in location from euchromatin to heterochromatin may have affected the molecular evolution of the gene. To this aim we performed a d_S_ - d_N_ tests, a codon-based test of selection (for details see material and methods), using DNA sequences from five representative *Yeti* genes: three located in heterochromatin (*Dmel*\*Yeti, Dsec*\*Yeti and Dere*\*Yeti*) and two in euchromatin (*Dpse*\*Yeti* and *Dvir*\*Yeti*). The results suggest that *Yeti* is under purifying (negative) selection when present both in heterochromatin and euchromatin. Thus, the change in genomic location does not appear to have affected significantly the molecular evolution/function of *Yeti* (Table 2).

**Table 2:** Codon-based test of selection.

Number of species compared	Number of codons^a^	d_S_ - d_N_	P^b^
5	232	10.2	<10^−6^
3 (heterochromatic sequences)	233	5.3	<10^−6^
2 (euchromatic sequences)	275	8.7	<10^−6^

a All positions containing gaps were eliminated.

b The probability (P) of rejecting the null hypothesis of strict-neutrality (d_N_ = d_S_) in favor of the alternative hypothesis (purifying selection, dN<dS) is shown.

## Discussion

In this paper, we have studied the evolutionary origin of *Yeti*, an essential gene of *Drosophila melanogaster* (Figure 1) located in the deep heterochromatin of chromosome 2 [25], [26], [28], [29] and found that it has evolved from a euchromatic ancestor in drosophilids.

Our FISH analysis shows that *Yeti* maintains a heterochromatin location in *2Rh*, at the base of division 41A, in both *D. simulans* and *D. sechellia* sibling species of *D. melanogaster* (Figure 2). The FlyBase localization of *Yeti* in *D. sechellia* scaffold_170:38,673..3,460), is in accord with our mapping results, while *D. simulans Yeti* is reported to map to the 3R arm (3,765..73,764; Table 1) in a 70 kb gene-poor genomic region. However, we are confident about the heterochromatin location of *Yeti* in *D. simulans* for the following reasons: First, as discussed in the result section, the FISH signal morphology produced by the *Yeti* probe is different from that usually seen with euchromatic probes and represents a distinctive mark for sequences derived from polytenized heterochromatin [30], [31]. Second, our FISH mapping of *Yeti* in *D. simulans* is based on three reproducible experiments, each carried out on several polytene chromosome figures obtained from at least 10 larvae. Finally, the paucity of genes around *D. simulans Yeti* is *per se* highly suggestive of heterochromatin localization. Thus, it is possible that the apparent discrepancy between our data and FlyBase may reflect an assembly error that occurred in the *D. simulans* genome sequence assembly, as reported by Schaffer et al. [32].

**Figure 8:**
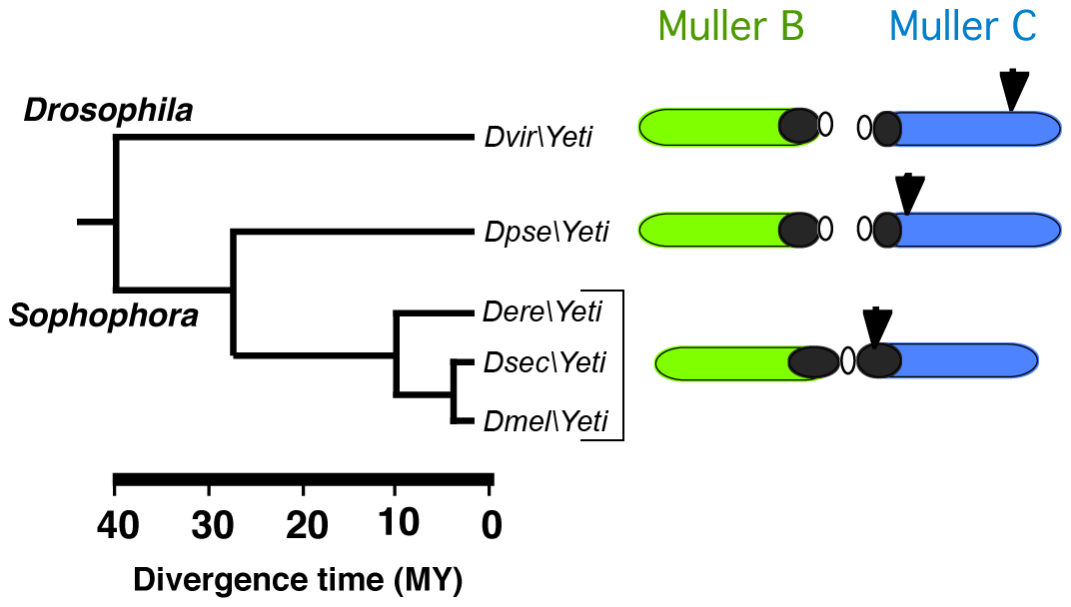
Evolutionary repositioning of the *Yeti* gene. Schematic representation of *Yeti* gene transition of from euchromatin to heterochromatin. The arrows point the chromosomal position of *Yeti*. It appears that *Yeti* has been resident on the Muller C chromosomal element, but over evolutionary time it progressively approached to pericentric heterochromatin and in *D. melanogaster* it is found in the deep portion of *2Rh*.

Our FISH analysis show that in two distantly related species, *D. pseudoobscura* and *D. virilis*, *Yeti* is located in euchromatin (Figure 2). In *D. pseudobscura, Yeti* maps to chromosome 3 at polytene division 63C, while in *D. virilis* it is found in chromosome 5, at polytene division 53E. Interestingly, *Yeti* lies in the syntenic chromosome Muller C that corresponds to the *2R* arm of *D. melanogaster* chromosome 2. Together, the results of our analysis indicate that during the evolution of the *Drosophila* genus, *Yeti* has been resident on the same chromosomal element, but over time it progressively moved closer to the pericentric regions. Such movements would have occurred in about 40 million of years, the estimated divergence time between *D. melanogaster* and *D. virilis* (Figure 8). A similar evolutionary trend was reported for *light* and other neighboring genes in *2L* heterochromatin [23] and for other genes of chromosome 3 heterochromatin [24].

A striking difference between heterochromatin and euchromatin genes lies in the generally larger size and complex molecular structure of the former compared to the latter. The example of the ‘giant’ Y-chromosome fertility factors of *D. melanogaster* mentioned above is paradigmatic in this respect [12]. Some of the essential heterochromatin genes of chromosomes 2 and 3 are also large due to the presence of large introns that harbour truncated TE copies (or TE “remnants”) [27], [35], [36], [37]. In this context, the *Yeti* gene of *D. melanogaster* with a 900 bp-long genomic region represents an exception [26]. The same is true for *RpL38, RpL5* and *RpL15*, three essential ribosomal protein-coding genes located in the heterochromatin of chromosomes 2 and 3, all of which are of relatively small size [24], [26], [37], [38]. How might these observations be explained?

One may imagine that, during evolution, genes increased their size by becoming targets for reiterated transposable-element insertions in the intronic regions, depending on their time of residence in heterochromatin. This, however, does not seem to have been the case. In fact, the *light* and *Yeti* genes, although having likely resided in heterochromatin for a comparable evolutionary time (less than 30 million of years), underwent a different molecular architecture; the *light* gene structure dramatically changed during the evolutionary transition from euchromatin to heterochromatin, due to a remarkable increase in the size of introns targeted by TEs [23]; *Yeti* retained its original organization in all analysed species, with a short genomic region carrying a single short intron (Figure 5). In addition, by PCR analysis we found that the *Yeti* intron does not undergo significant interspecies or intraspecies changes of its physical size (Figure 6). Similarly, the *RpL15* gene shows a conserved structure among *Drosophila* species, independently from its genomic location [24].

How to explain the different behaviour of heterochromatin genes? In particular, there might have been a selective pressure to maintain some genes of short size (with few, short introns) despite of their genomic location, owing to their particular functional requirement: interestingly in that respect, highly expressed genes are known to harbour substantially shorter introns than genes that are expressed at low levels [39]. This may be the case of *Yeti* and *RpL38*, *RpL5* and *RpL15* heterochromatic genes of *Drosophila melanogaster*, which are all highly expressed and all have indeed short size and carry short introns [26], [37], [38]. *Yeti* itself encodes an important chromatin-remodeling factor required for development [26] and ribosomal protein coding genes are also essential for proper development. It is not unreasonable to speculate that these genes maintained the original structure, in spite of their transition to heterochromatin during massive chromosomal rearrangements that occurred over time, because of the requirement for their efficient expression during early development. In addition, it is possible that once in heterochromatin, a given sequence might be differentially targeted by transposable elements, with some sequences being more refractory than others. These observations suggest that the evolutionary forces that acted in shaping the structural organization of genes currently found in *D. melanogaster* heterochromatin are molecularly diverse.

Finally, the results of our d_S_ - d_N_ tests, showing that *Yeti* is under negative selection both in heterochromatin and euchromatin (Table 2), are in accord with its evolutionary conserved function and suggest that the change in genomic location did not affected significantly the molecular evolution of the gene.

Together, these results contribute to expand our understanding of the molecular dynamics driving the evolution of the heterochromatin genome in higher eukaryotes.

### Materials and Methods

### Drosophila strains

Fly cultures were carried out at 25°C in standard cornmeal yeast medium. *D. melanogaster* strains derived from natural populations: Altamura I and Altamura II (South Italy), Beijing (Cina), California and California I (USA), Charolles (France), Charolles 1999 (France), Friburgo 1997 (Germany), HJ30 (France), Hobo (Grece) Israel-4 (Israel), Lanuvio (Italy), Luminy (France), Mali and Mali I (West Africa), Marrakesh (North Africa), Scansano (Central Italy), Vallecas (Spain), W15 (USA), W30 (USA), W90 (USA), W130 (USA), W135 (USA), Zimbabwe (Africa), Gaiano (North Italy). *D. melanogaster* laboratory strains (separated by comma): isogenized *y^1^; cn^1^,bw^1^,sp^1^* strain (Iso) [40]. *l(2)LP2/SM1,Cy*. *Cy/Sp;Sb,Delta2-3,ry^506^/TM6,ry^506^*. *l(2)EMS-31/SM1,Cy*. *D. simulans* strains derived from natural populations: Armidale (New South Wales, Australia), Canaries (Atlantic Spain), Can River (Australia) Chantal (France), Chicharo (Portugal), Death Valley (USA), Genoa (Italy), Kyogle (Australia) and Moruya (New South Wales, Australia). *D. melanogaster* and *D. simulans* wild-type strains were derived from natural populations collected in the wild before the end of year 2000 and are gifts of Sergio Pimpinelli, Nikolaj Junakovic, Chantal Vaury and Pierre Capy.

### Cytology and fluorescent in situ hybridization

Polytene chromosomes prepared according to Pardue [41] were stained with DAPI. The *D. melanogaster* RE36623 cDNA *Yeti* probe was labelled by nick-translation with Cy3-dCTP (Amersham). Species-specific PCR probes were used for FISH in D. pseudobscura and D. virilis (see below). FISH procedures were performed according to Dimitri [42]. Digital images were obtained using an Olympus epifluorescence microscope equipped with a cooled CCD camera. Gray scale images, obtained separately recording Cy3 and DAPI fluorescence by specific filters, were pseudo colored and merged for the final image using the Adobe Photoshop software.

### Nucleic acid manipulation and sequence alignments

Genomic DNA extraction and PCR were performed according to the protocol of Berkeley Drosophila Genome Project (http://www.fruitfly.org/about/methods/index.html). To PCR amplify *Yeti* genomic fragments from *D. melanogaster, D. simulans* strains, *D. sechellia* and *D. tessieri* the following primers were used: F: 5′-TTAGTATGGCCTGCGAGACA-3′; R: 5′-TGTGCTCGCATAACAAAGGC-3′.

PCR cycle were: 40″ at 98°C, 40 x (10″ 98°C, 30″ 58°C, 8″ 72°C). Amplified fragments were gel purified and sequenced by Bio-Fab research s.r.l. The *Yeti* probes from *D. pseudoobscura* and *D. virilis* were generated by PCR over genomic DNA with the following primers:

Dpse-F 5′-GCGACGATGATAGCATCAAT-3′; Dpse-R 5′-GTGAGTGCTCAGCTGCTCAT-3′

Dvir-F 5′-AGCTAAACGTAGCACGCGTC-3′; Dvir-R 5′-TGTGTACGCAGATCCTCGTC-3′

PCR cycle were: 4′ at 94°C, 35 x (30″ 95°C, 45″ 60°C, 30″ 72°C). Amplified fragments were cloned in pGEM-T vector (Promega) and verified by DNA sequencing.

Multiple sequence alignment were performed by ClustalW procedure available at EMBL-EBI (http://www.ebi.ac.uk) or with the multialin interface at http://multalin.toulouse.inra.fr.

### Testing for signatures of selection

A codon-based test of selection was conducted in MEGA 6.0 [43], using the Nei-Gojobori method [44]. The statistic test (d_S_ – d_N_) is expected to be zero under the null hypothesis of neutrality. d_S_ and d_N_ are the numbers of synonymous and nonsynonymous substitutions per site, respectively. Three analyses were performed. The first analysis involved five coding sequences of the *Yeti* gene from five different Drosophila species (*D. melanogaster*, *D. simulans*, *D. erecta*, *D. pseudoobscura* and *D. virilis*). The other two analyses involved either the three Drosophila species where the *Yeti* gene is heterochromatic (*D. melanogaster*, *D. simulans* and *D. erecta*) or the two species where the *Yeti* gene is euchromatic (*D. pseudoobscura* and *D. virilis*).
